# Developing a proactive coping theory-based conceptual framework for sarcopenia management in aging societies: a mixed-methods study from China

**DOI:** 10.3389/fpubh.2025.1604370

**Published:** 2025-11-20

**Authors:** Ruiqi Dai, Xinqun Feng, Yixi Weng, Lei Mao

**Affiliations:** 1 College of Fashion and Design, Donghua University, Shanghai, China; 2 School of Design, Nanjing University of the Arts, Nanjing, China; 3 College of Fine Art, Nanjing Normal University, Nanjing, China; 4 Design School, Xi’an Jiaotong-Liverpool University, Suzhou, China; 5 Institute of Population Health, University of Liverpool, Liverpool, United Kingdom

**Keywords:** muscle energy attenuation, sarcopenia, system design intervention, proactive coping theory, design strategy, aging society

## Abstract

**Introduction:**

This study proposes a public health strategy to combat sarcopenia in rapidly aging societies, addressing systemic gaps in preventive healthcare through a proactive, design-driven framework.

**Methods:**

A mixed-methods approach was adopted, integrating participatory questionnaires (*n* = 1,683) and grounded theory–analyzed interviews (*n* = 48). Stage classification was validated through triangulation of self-reported activity limitations, clinimetric scoring, and biomechanical assessments. The Analytic Hierarchy Process was employed to decode dynamic weighting mechanisms among physiological determinants, psychological factors, and fixed environmental parameters.

**Results:**

The study constructed a self-evaluated four-stage progression model applicable to urban contexts. By bridging clinical diagnostics with daily life narratives, the framework enables earlier risk identification outside healthcare settings. A novel mapping algorithm was devised, correlating patient-reported disease staging with evidence-based intervention tiers. The resultant three-tier system operationalizes cognitive reframing and behavioral reconfiguration mechanisms, aligning patient self-assessment with targeted intervention design.

**Discussion:**

This interdisciplinary model synergistically addresses three critical objectives: healthcare resource optimization, social participation longevity enhancement, and disability trajectory modulation. By positioning design as an ecological mediator, the framework supports the transition of healthcare systems from acute care paradigms to preventive ecosystem orchestration, ultimately fostering equitable health resilience within aging societies.

## 1 Introduction

China’s accelerating population aging presents unprecedented public health challenges. The nation’s demographic transition exhibits two distinctive characteristics: First, a wide scale, as, according to the latest 2024 data from China’s National Bureau of Statistics, the population aged over 65 has reached 220 million, accounting for approximately 18.3% of the global older adults population (1); Second, rapid transition velocity, as China has transitioned from an aging to a super-aged society within 23 years (2). This rapid aging amidst resource constraints creates systemic pressures: healthcare gaps between urban and rural regions and delayed management of age-related chronic conditions.

Within this aging context, sarcopenia emerges as a critical intervention target given its high prevalence and preventable nature among degenerative conditions. The pathogenesis of sarcopenia involves multifactorial dysregulation: Age-related denervation preferentially triggers type II fiber atrophy, compounded by impaired satellite cell regeneration, metabolic imbalance, chronic inflammation, anabolic hormone decline, and mitochondrial dysfunction, collectively driving progressive muscle deterioration (3, 4). While China’s 2024 health policy elevates older adults chronic disease management through increased public health funding (reaching 94 yuan per capita) (5), standardized sarcopenia screening protocols and scalable community-level interventions remain critically underdeveloped. One study revealed that China’s sarcopenia prevalence stands at 17.4% among those aged over 65, showing a significant upward trend with a 2.7% increase from the period 2014–2017 to the period 2018–2021. The condition demonstrates marked age correlation, with prevalence rates of 9.1, 18.0, and 38.1% in the 65–69, 70–79, and over 80 age groups, respectively (6). Sarcopenia is pathologically characterized by progressive skeletal muscle mass reduction, leading to decreased strength and physical function (termed muscle attenuation syndrome) (7). As the lower limb muscles bear body weight, they exhibit earlier and faster deterioration. After the age of 50, leg muscle mass declines by 1–2% annually, accompanied by 1.5–5% annual strength loss (8). This muscular degeneration manifests in standing difficulties, balance impairment, and eating tremors, directly compromising older adults autonomy and constituting a critical factor in late-life quality (9).

Sarcopenia intrinsically drives severe complications in older adults, including elevated fall and fracture risks, and strong associations with cardiovascular diseases, respiratory disorders, and cognitive impairment (10). Paradoxically, this high-risk condition remains underdiagnosed in clinical settings due to misconceptions of its non-immediate mortality risk, leading to widespread undertreatment. Consequently, neglected sarcopenia exacerbates healthcare burdens through significantly elevated hospitalization rates, prolonged recoveries, and increased medical expenditures which ultimately compounding individual health risks and systemic costs.

These compounding burdens highlight an urgent need for systematic interventions to mitigate the cascading impacts of sarcopenia. Given this context, establishing comprehensive life cycle health management systems for sarcopenia within aging populations could prove vital—not only for reducing preventable healthcare expenditures but also for enhancing sustainable well-being in late life.

## 2 Theoretical framework

### 2.1 Systematic assessment of sarcopenia related products and stratified targeting strategy

We cataloged commercially available sarcopenia related products to establish the current market landscape. Existing evidence indicates that rural older adults exhibit markedly lower adoption rates of existing assistive products (11) shown in Table 1 (excluding canes) due to constrained educational attainment and economic constraints (12). These factors collectively impede the uptake of both conventional and innovative interventions in rural settings. Conversely, research suggests a contrasting functional profile: sustained engagement in agricultural activities preserves rural elders’ physical autonomy (13), whereas urban counterparts, despite greater nutritional diversity, face elevated disability risks associated with dietary patterns, exhibit excessive intake of high-processed foods correlating with health risk (14).

**Table 1:** Commercially available sarcopenia intervention products.

Type	Product	Image	Target area	Primary user groups
Mobility assistance walking aid	Rollator	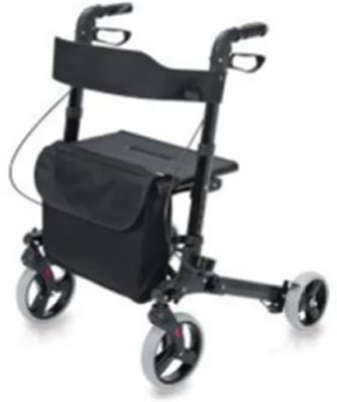	Lower limbs (Hip/Knee/Ankle)	Geriatric population
	Cane	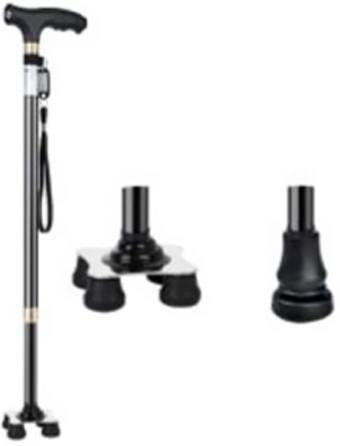	Upper limb support/Load reduction	Individuals with lower limb dysfunction; Geriatric population; Patients in recovery phase
	Wheelchair	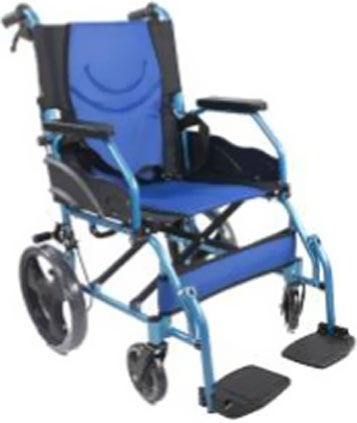	Full-body mobility compensation	Individuals with lower limb dysfunction; Geriatric population; Patients in recovery phase
Joint protection & fall prevention	Knee brace	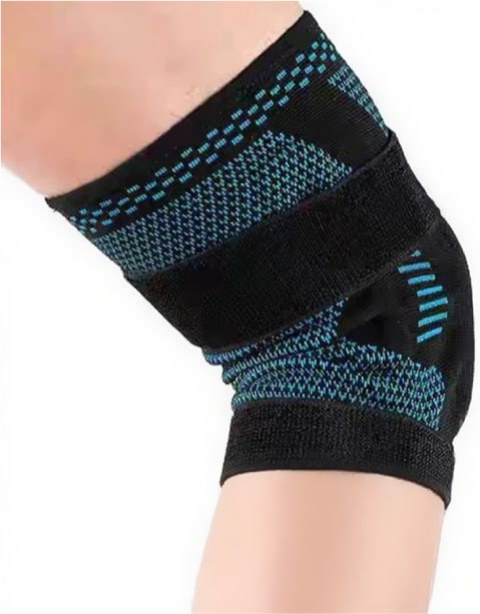	Knee joint	Athletes; Middle-aged and older adults; Individuals with knee pathologies
	Hip protector	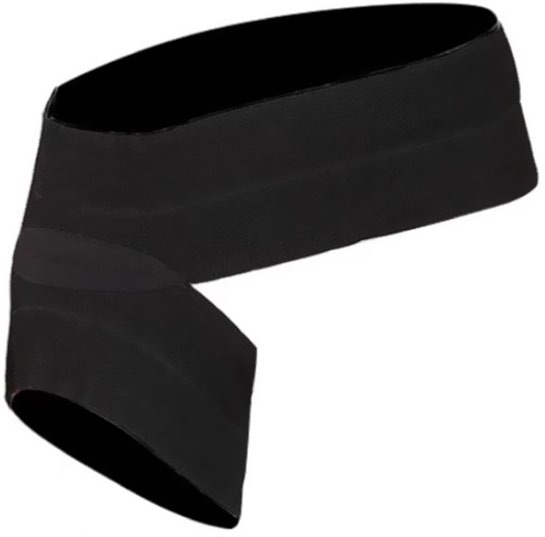	Hip impact absorption	Osteoarthritis patients require: • Stabilization • Thermal protection
	Anti-slip footwear	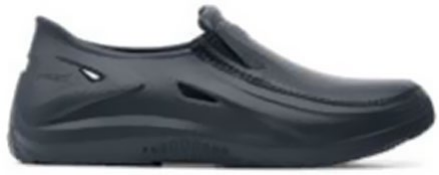	Foot friction enhancement	outdoor workers; Geriatric population; Pregnant individuals and clinical personnel
Rehabilitation training & muscle activation	Resistance band	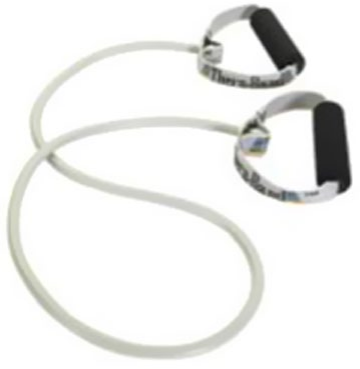	Full-body muscle activation	Fitness enthusiasts; Rehabilitation patients; Geriatric population
	Grip strengthener	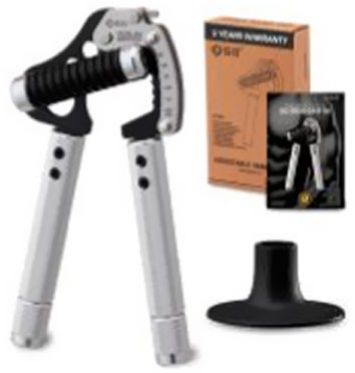	Forearm & hand muscles	Fitness enthusiasts; Rehabilitation patients; Sedentary Workers; Performance Athletes; Active Maintainers
	Balance training mat	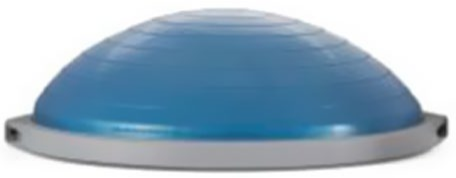	Lower limb training	Fitness enthusiasts; Rehabilitation patients; Children; Sedentary Workers
Smart monitoring & emergency alert	Wearable fall detector	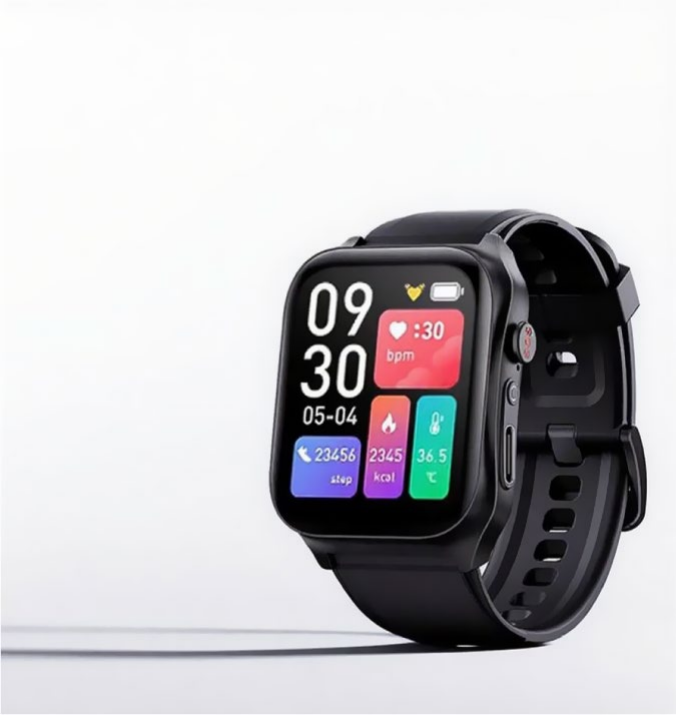	Full-body monitoring	Seniors living alone
	Intelligent gait analysis sensor	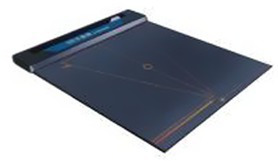	Lower limb gait analysis	Hospital; rehabilitation institutions
	Muscle mass analyzer	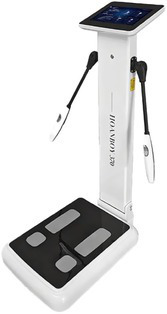	Full/regional muscle assessment	Medical institutions; Premium fitness centers

All visual data presented were sourced exclusively from Taobao, China’s leading e-commerce platform. Product descriptions and target user groups are compiled by the authors based on common features and intended uses of commercially available products in this category.

Micro-level product deficiencies manifest in three aspects: 1. Cross-cultural contextual conflicts, with North American-dominated rollator designs presuming detached housing, conflicting with China’s high-density apartments. In older communities with no access to elevators, rollators become impractical due to inaccessible staircases. 2. Preventive health cognition gaps, resistance bands and balance mats developed from sports medicine principles remain categorized as “fitness equipment” in public perception. Despite their preventive efficacy against sarcopenia, target users show low adoption willingness (15). 3. The public-private technological disconnect. Advanced devices, such as intelligent gait analysis sensors and muscle mass detectors, primarily serve institutional users with higher costs, rarely penetrating consumer markets.

Macroscopically, unlike acute conditions such as sprains or strokes, sarcopenia follows a predictable vicious cycle: progressive muscle loss, strength decline, functional disability, and ensuing complications. Yet current market offerings predominantly provide passive responses and target isolated symptoms using modularized solutions, such as grip strengtheners for grip weakness but fail to systematically address disease progression. This product–pathology disjunction stems from dual neglect: insufficient understanding of sarcopenia’s dynamic characteristics within rehabilitation design, and disregard for users’ behavioral plasticity to interrupt disease progression.

Therefore, our design strategy fundamentally shifts from reactive symptom management to a proactive paradigm aimed at preempting sarcopenia’s pathological progression before significant functional decline, thereby achieving sustainable disability mitigation.

### 2.2 Theoretical foundations: from positive psychology to proactive coping theory

To operationalize this proactive intervention paradigm addressing Section 2.1’s reactive approach limitations, we ground our design strategy in Proactive Coping Theory (PCT). As an emerging focus within positive psychology, the definition of Proactive Coping Theory is to evaluate future objectives and establish the necessary conditions for their successful achievement (16). It emphasizes preparing for anticipated stressors rather than reacting to present adversities (17). This theory frames proactive coping as future-oriented self-regulation through goal appraisal and preparatory actions (18), thereby directly aligning with our objective of enabling users to preemptively manage sarcopenia risk and mitigate progression.

Internationally, design applications of positive psychology concentrate on Positive Design, notably Desmet (19) from Delft University of Technology’s framework of prioritizing human flourishing through the dimensions of pleasure, meaning, and virtue. Hassenzahl (20) from Folkwang University of the Arts advanced experience-driven approaches, conceptualizing material–cultural interactions for meaningful pattern generation.

In China, Positive Experience Design focuses on emotional optimization strategies. Professor Wu (21) from Donghua University proposed shared product algorithms to enhance user happiness, as detailed in *Packaging Engineering*. Dr. Liu (22) from Wuhan Textile University systematized a four-phase affective design, incorporating contextual needs, sensory instincts, functional interactions, and reflective experiences.

While contributing methodological insights, these approaches differ fundamentally from Proactive Coping Theory as they prioritize emotional activation over cognitive-behavioral interventions. The “Seed Currency” mechanism at the Dandelion older adults Rehabilitation Center (Aichi-ken, Japan) exemplifies proactive capacity-building through value restoration designs (23), providing critical referential value.

Consequently, this study synthesizes PCT with sarcopenia intervention in order to achieve two synergistic advances: First, it transforms conventional reactive, reparative, and symptomatic approaches into proactive, preventive, and systematic methodologies, fundamentally reconfiguring intervention paradigms. Second, by proposing a systemic life cycle-based design framework, we explored interdisciplinary applications of Proactive Coping Theory for chronic disease management designs for aging populations, extending Positive Psychology’s functional scope beyond conventional emotional optimization.

### 2.3 Interdisciplinary pathways: proactive coping theory-driven muscle attenuation interventions

While symptom-specific clinical diagnostic criteria and therapeutic protocols for sarcopenia have achieved relative maturity (24), as evidenced by the <2024 Chinese Sarcopenia Diagnosis and Treatment Guidelines> targeting older adults patients with suspected or confirmed diagnoses, China’s national healthcare insurance system conspicuously excludes preventive sarcopenia screening from reimbursement coverage. This institutional gap fundamentally conflicts with sustainable public healthcare.

Also, current interventions remain pathologically reactive due to two critical limitations: Firstly, the European Working Group on Sarcopenia in Older People (EWGSOP) categorizes sarcopenia progression into three stages: pre-sarcopenia, sarcopenia, and severe sarcopenia. However, this classification system exhibits arbitrary thresholds that lack standardized diagnostic criteria (10), with assessment parameters demonstrating ethnic variability across populations (6). Consequently, patient-reported outcomes may supersede biomedical metrics in terms of clinical relevance.

Secondly, although historically conflated with aging, sarcopenia pathogenesis initiates during midlife (25). Therefore, early interventions significantly mitigate progression (26). Current medical detection prioritizes symptomatic patients using the following: 1. the SARC-F questionnaire, its adoption is limited in primary care settings due to its exclusion from national health screening protocols; 2. Ishii screening, which was developed for Japanese septuagenarian cohorts (27) and has uncertain validity for younger demographics; 3. clinical imaging, including dual-energy X-ray absorptiometry (DXA) and bioelectrical impedance analysis (BIA), which are deployed after the manifestation of symptoms (28).

Existing sarcopenia interventions remain pathologically reactive due to their symptom-centric focus. To clearly translate the core elements of Proactive Coping Theory into a design framework for sarcopenia management, this study employs the five-stage process model proposed by Aspinwall and Taylor (18) as a guiding structure. The specific application of these five stages is detailed as follows:

Stage 1: Resource Accumulation. Theoretically, this involves the preemptive accumulation of resources, such as knowledge, skills, and social support, necessary to address future challenges. Applied to sarcopenia management, it necessitates breaking down information barriers to raise disease risk awareness, particularly among younger demographics, and establishing a foundation of knowledge and skills for self-management. This directly aligns with our core principle of proactively shifting intervention focus to midlife pathogenesis, empowering individuals to prioritize functional capacity preservation over disease treatment, thereby effectively reducing disability risk (29, 30).
Stage 2: Recognition of Potential Stressors. The theoretical core lies in proactively identifying future events that may pose challenges. Within sarcopenia management, the key is guiding individuals aged 40–60 to reframe muscle attenuation as a manageable health challenge rather than an inevitable consequence of aging. Crucially, this stage empowers patient-initiated risk identification through the use of perceptible functional indicators for streamlined screening. This approach thereby overcomes the institutional screening barriers associated with the SARC-F questionnaire and the demographic biases inherent in the Ishii screening tool, significantly reducing reliance on costly DXA testing.
Stage 3: Initial Appraisal. This stage entails cognitively assessing the identified stressor to evaluate its controllability and available coping resources. For sarcopenia management, users assess three critical dimensions: their personal risk severity, agency in managing the condition and available coping resources, such as family support and economic capacity. This appraisal underpins personalized intervention strategy formulation.
Stage 4: Preliminary Coping Efforts. Based on the appraisal results, this stage involves initiating concrete preparatory actions. In sarcopenia management, this translates to guiding individuals to implement preventive behavioral interventions, transforming cognitive awareness into early action.
Stage 5: Elicitation and Use of Feedback. This stage focuses on monitoring initial effort effects, evaluating effectiveness, and making dynamic adjustments. Applied to sarcopenia, it requires tracking behavioral adherence and functional indicator changes, providing real-time feedback to reinforce positive behaviors, identify barriers, and optimize plans. The ultimate goal is establishing a sustainable self-management feedback loop for ongoing intervention efficacy.

## 3 Materials and methods

Given the trifecta of the public awareness gap, insufficient primary care diagnostics, and chronic disease latency, sarcopenia’s diagnostic rates significantly under-represent its actual prevalence. Therefore, our design practice began with the identification of confirmed and potential sarcopenia patients via dynamic stratified sampling.

### 3.1 Study population

Section 2.1 evidences lower adoption rates of sarcopenia interventions in rural areas versus urban settings, coupled with elevated sarcopenia risk among urban older adults. This urban–rural dichotomy reveals systemic healthcare inequities in aging populations, necessitating interdisciplinary, design-driven solutions. To maximize accessibility and diffusion potential, our strategy prioritizes older adults residents in China’s first and second-tier^1^ cities as primary research samples and initial beneficiaries. Future rural scalability is anticipated through reverse migration patterns of urban workers returning to hometowns.

### 3.2 Preliminary screening through questionnaire

Having established this urban-focused cohort, we implemented a culturally-adapted preliminary screening protocol to identify sarcopenia risk trajectories. The Sarcopenia Self-Assessment Questionnaire shown in Table 2 translates abstract metrics into tangible life symbols in China. For example, it replaces “5 kg” with oil-lifting tests that establish mass-muscle correlations and it conceptualizes walking distances using “market–delivery station” and “residential complex-living room” spatial analogies. This lifestyle-embedded screening mechanism enables early non-clinical detection via self-assessed physical performance. The scale categorizes 0–8 points as “Healthy,” 9–16 points as “Pre-Sarcopenia Compensation,” 17–24 points as “High Risk,” and ≥ 25 points as “Disabling Degeneration.” Questionnaires were disseminated through traceable community networks, encompassing both digital and physical channels.

**Table 2:** Sarcopenia self-assessment questionnaire.

Assessment domains	Items	Question	0 points	1 point	2 points	3 points
Strength performance	1	How do you usually go up/down stairs?	Go up/down without holding the railing	Need to lightly touch the railing for balance	Put both feet on each step	Need someone to help
	2	Can you lift a 5 L cooking oil bottle to table height?	Easily lift with one hand	Need both hands to lift	Have to press it against my belly to lift	Cannot do it at all
	3	When getting up from a chair with no armrests:	Stand up without using hands	Push on my knees to stand up	Need to hold something nearby	Need someone to pull me up
Endurance capacity	4	What’s the farthest you can walk non-stop?	Walk to a market 1 km away	Reach the delivery station at the neighborhood	Only walk around the garden downstairs	Get tired after walking across the living room
	5	What chores can you do while standing?	Mop all rooms without stopping	Only mop one room	Have to sit while prepping veggies/folding clothes	Cannot do chores alone
	6	How would you hang 10 pieces of laundry?	Finish all at once without breaks	Do it in two rounds	Need to sit and rest during the task	Cannot do it by myself
Balance regulation	7	Can you put on lace-up shoes with one foot?	Balance on one foot easily	Need to lightly hold a table	Must keep both feet on the ground	Need help from others
	8	How do you walk on rainy days?	Walk normally	Take small careful steps	Need someone to hold me	Avoid going out
	9	When avoiding obstacles (like bikes/potholes):	Quickly step sideways to dodge	Stop and wait for it to pass	Stagger and grab something	Might lose balance
Functional adaptation	10	How do you shop at supermarkets?	Carry a basket to checkout	Must use a shopping cart	Need staff to help bag items	Only shop online
	11	How do you reach something slightly above your head?	Stand on tiptoes to grab it	Use a stool to reach	Ask someone to steady me	Give up and leave it
	12	How do you stand on public buses?	Stand firmly anywhere	Move near handrails first	Lean against the wall	Must sit down, avoid rush hours

### 3.3 Secondary screening through symptom-behavior correspondence framework

**Figure 1:**
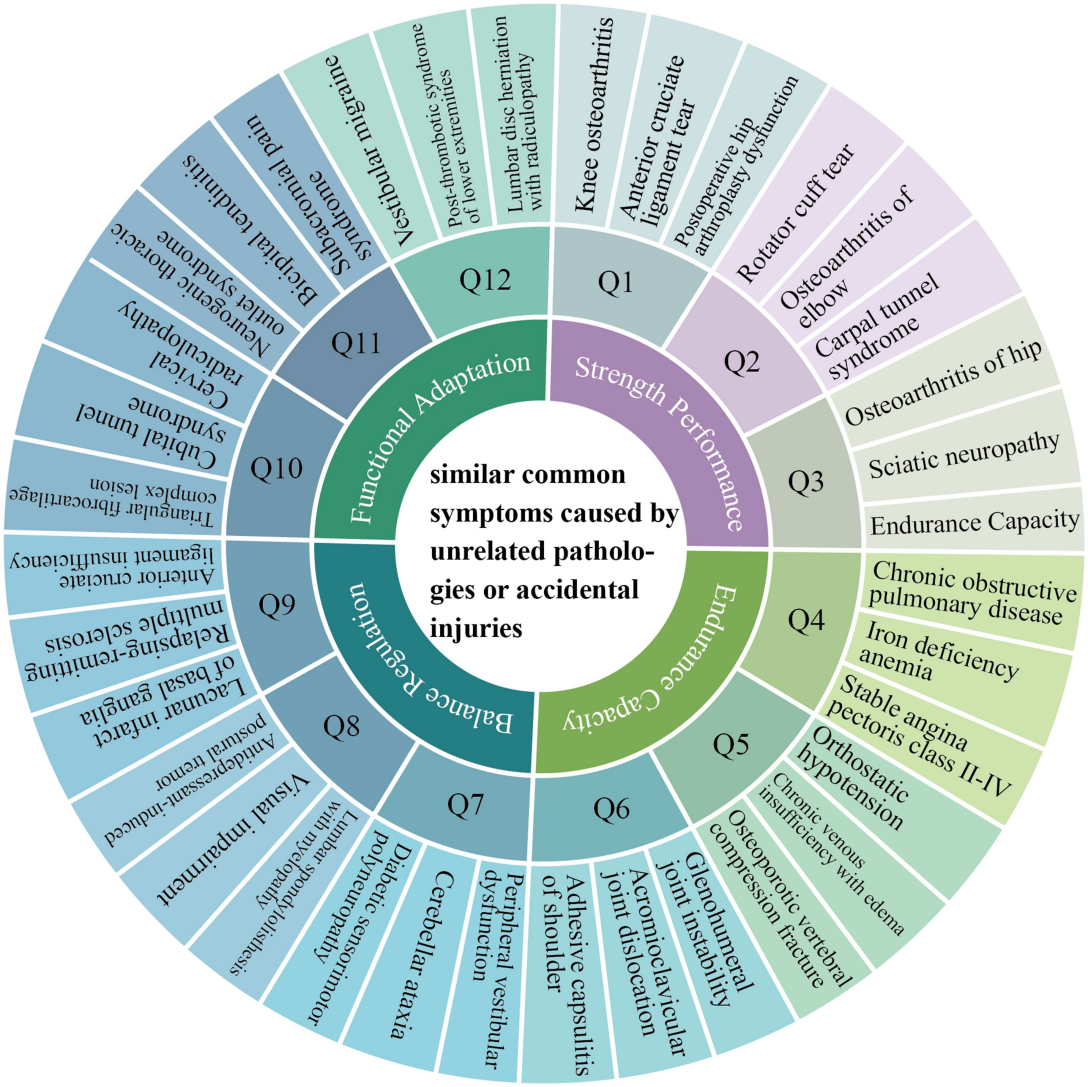
Similar symptoms caused by unrelated pathologies or accidental injuries (draw by author).

The secondary screening implemented a symptom-behavior correspondence framework to exclude participants exhibiting sarcopenia-like manifestations from unrelated pathologies or trauma (Figure 1).

This process accommodated participants’ intentional disease reporting vagueness, wherein 765 individuals with clinically confirmed diagnoses self-identified using broad pathological categories, such as reporting “heart disease” rather than specifying hypertrophic cardiomyopathy. This obfuscation primarily stemmed from cultural privacy preservation norms wherein participants guarded detailed health information from non-medical personnel. Crucially, as disease classification fell beyond this sarcopenia-focused investigation’s scope, interviewers deliberately avoided diagnostic probing to respect autonomy. Concurrently, 185 symptomatic undiagnosed individuals described non-specific complaints such as “chronic low back pain restricting movement,” “unsteady walking without known cause.”

As systematically tabulated in Table 3, our exclusion criteria specifically targeted pathologies capable of mimicking sarcopenic phenotypes through three primary mechanistic pathways: mechanical interference from joint diseases (*n* = 475) inducing joint pain, reduced activity, muscle atrophy (31); metabolic interference arose predominantly from endocrine conditions (*n* = 161) disrupting essential protein anabolism pathway and oxygen transport deficiency; while neurological confounding in relevant disorders (*n* = 91) manifesting clinically significant strength and balance deficits (32). Additionally, the “Other conditions” category (*n* = 16) encompassed clinically relevant transient states including activity-induced myalgia, exemplified by reports of generalized soreness following prolonged childcare, and traumatic mobility limitations such as those described in cases of recent ankle injury significantly limiting ambulation capacity.

**Table 3:** Participants excluded due to unrelated pathologies.

Exclusion condition category	Confirmed cases	Symptomatic undiagnosed	Total	Confounding factors with sarcopenia
Joint diseases	380	95	475	Joint pain, reduced activity, muscle atrophy
Metabolic/endocrine disorders	126	35	161	Impaired protein synthesis, oxygen transport deficiency
Neurological disorders	76	15	91	Decreased muscle strength, gait instability, balance impairment
Cardiovascular diseases	66	14	80	Reduced exercise tolerance, fatigue, muscle weakness
Chronic respiratory diseases	39	7	46	Exertional dyspnea, decreased physical activity
Postoperative recovery	43	0	43	Delayed recovery of muscle strength post-rehabilitation
Visual system disorders	11	10	21	Visual impairment, balance dysfunction
Inflammatory/autoimmune diseases	15	2	17	Restricted joint mobility
Other conditions	9	7	16	Reduced activity, mobility limitation

Post-removal of 950 confounding cases, application of the 9-point diagnostic threshold excluded 83 additional subjects, establishing a rigorously defined cohort of 650 participants for sarcopenia-specific assessment (Table 3).

### 3.4 Profiling through in-depth interview

The third step was the in-depth profiling layer, during which a stratified disease progression evaluation system was developed, incorporating semi-structured qualitative interviews with 48 consenting participants, whose age distribution is detailed in Table 4.

**Table 4:** Baseline characteristics of study participants.

Age	Phase	Gender (Male:Female)	Years of education	Health insurance type	Monthly income (CNY)
<30	Scr (*n* = 281)	145:136	13.5 ± 3.5	StuIns: 65%; EmpIns: 25% ResIns: 10%	2,500 ± 1800
31–40	P-Scr (*n* = 171)	89:82	14.2 ± 2.8	EmpIns: 78%; ResIns: 19% NoIns: 3%	12,800 ± 4,500
	S-Scr (*n* = 28)	15:13	12.8 ± 2.6	EmpIns: 71%; ResIns: 29%	11,500 ± 4,000
	Prof (*n* = 2)	1:1	12.5 ± 2.8	EmpIns: 100%	11,500–13,000
41–50	Scr (*n* = 274)	152:122	12.1 ± 3.1	EmpIns: 75%; ResIns: 23% NoIns: 2%	11,200 ± 4,000
	S-Scr (*n* = 129)	67:62	11.2 ± 3.0	EmpIns: 68%; ResIns: 28% NoIns: 4%	10,000 ± 3,500
	D-Prof (*n* = 8)	4:4	10.8 ± 3.2	EmpIns: 75%; ResIns: 25%	11,400 ± 2000
51–60	P-Scr (*n* = 344)	188:156	10.3 ± 3.4	EmpIns: 65%; ResIns: 25% NRCMS: 8%; NoIns: 2%	8,500 ± 3,000
	S-Scr (*n* = 138)	72:66	9.5 ± 3.2	EmpIns: 58%; ResIns: 30% NRCMS: 10%; NoIns: 2%	8,900 ± 2,500
	Prof (*n* = 15)	8:7	8.9 ± 3.0	EmpIns: 53%; ResIns: 35% NRCMS: 10%; NoIns: 2%	9,300 ± 2,200
61–70	Scr (*n* = 447)	241:206	8.7 ± 3.8	EmpIns: 48%; ResIns: 35% NRCMS: 15%; NoIns: 2%	5,500 ± 2000
	S-Scr (*n* = 257)	132:125	7.9 ± 3.5	EmpIns: 42%; ResIns: 40% NRCMS: 16%; NoIns: 2%	5,800 ± 1800
	D-Prof (*n* = 18)	9:9	7.2 ± 3.1	EmpIns: 39%; ResIns: 45% NRCMS: 15%; NoIns: 1%	4,600 ± 2000
71–80	P-Scr (*n* = 103)	63:40	6.5 ± 4.1	EmpIns: 30%; ResIns: 45% NRCMS: 23%; NoIns: 2%	3,500 ± 1,200
	S-Scr (*n* = 98)	51:47	6.0 ± 3.8	EmpIns: 28%; ResIns: 48% NRCMS: 22%; NoIns: 2%	3,200 ± 1,000
	Prof (*n* = 5)	3:2	5.8 ± 3.5	EmpIns: 100%	3,000–5,000
>81	Scr (*n* = 63)	24:39	5.1 ± 4.3	EmpIns: 85%; NRCMS: 15%	2,800 ± 800

P-Scr, preliminary screening; S-Scr, secondary screening; D-Prof, in-depth profiling; StuIns, Student Health Insurance; EmpIns, Employee Basic Medical Insurance; ResIns, Urban–Rural Resident Insurance; NRCMS, New Rural Cooperative Scheme; NoIns, uninsured.

The interview protocol comprises four sections. Section one is collecting demographic profile, including residential type (living alone/family), and marital status. Sections two to four constitute the core thematic domains for in-depth exploration, encompassing: Illness perception and disease trajectory; functional limitations and compensatory mechanisms; intervention needs and current barriers. The qualitative analysis followed grounded theory methodology.

### 3.5 Objective intervention stratification through AHP framework

Fourth Step, to achieve the transition from the qualitative stage model to objective intervention grading, this study developed a procedural mapping algorithm. Our proposed intervention framework adopted a proactive prevention-oriented approach, structured around sarcopenia’s pathological progression patterns, to establish a three-tiered hierarchical intervention strategy (Grades I, II, and III) calibrated to severity levels.

**Figure 2:**
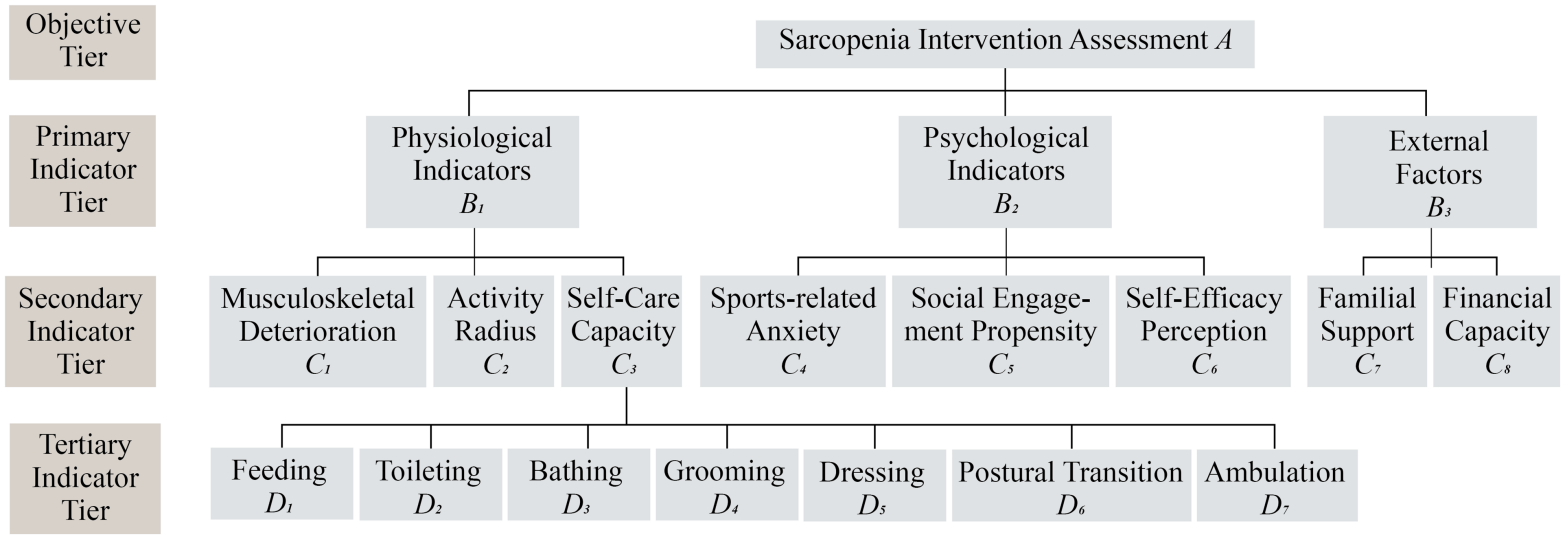
AHP of the muscle loss intervention (draw by author).

Building upon the Analytic Hierarchy Process (AHP) framework, we established a stratified evaluation system comprising three primary indicator strata which are physiological, psychological, and external factors, they were further subdivided into nine secondary indicators and seven tertiary indicators (Figure 2). Weight allocations integrated expert evaluations from 10 specialists at the Third Affiliated Hospital of Fujian University of Traditional Chinese Medicine with functional independence metrics derived from the Activities of Daily Living (ADL) scale, a clinically validated tool particularly relevant for sarcopenia assessment, given that sarcopenia doubles ADL disability risk in older adults populations (33).

## 4 Results

### 4.1 The result of the questionnaire

A total of 1728 participants completed the survey. After excluding individuals with missing data (*N* = 45), the final analytical sample comprised *N* = 1,683 subjects. Prior to empirical analysis, reliability and validity tests of the scale were conducted. This study employed Cronbach’s *α* coefficient to examine the questionnaire’s reliability. Results indicated that the overall scale demonstrated excellent internal consistency (α = 0.89). All subscales showed α = 0.78–0.85, exceeding the recommended threshold of 0.70. Detailed results are presented in Supplementary Table S1.

Subsequent construct validity testing employed factor analysis with Kaiser-Meyer-Olkin (KMO) sampling adequacy assessment. As shown in Supplementary Table S2, Bartlett’s test of sphericity was statistically significant (*p* < 0.001), satisfying the sphericity assumption. The combination of KMO measure and Bartlett’s test results established the suitability of the data for factor analysis.

Building upon these validated measures, age-stratified profiles emerged (Table 4). Individuals under 30 years of age demonstrated healthy physical parameters. Within the 31–40 year-old cohort, the average assessment score was 8.2, approaching the pre-sarcopenia threshold, with postural stability showing measurable decline. Notably, this group exhibited a 38% increase in obstacle avoidance failure rates compared to their younger counterparts. The 51–60 age demographic manifested tripartite stratification: 42.9% maintained healthy status, while 28.6% presented pre-sarcopenia indicators and 28.5% demonstrated high-risk profiles. A critical inflection occurred in the 61–70 cohort, where risk parameters escalated exponentially, aligning with established sarcopenia onset patterns. All sampled individuals in this group exceeded the pre-sarcopenia threshold, as evidenced by a 210% deterioration in stair negotiation performance compared to preceding age groups. Severe functional impairments emerged in subsequent age groups, with 95% of the 71–80 year olds requiring assistance for sit-to-stand transitions, while 75% needed mobility support during inclement weather. The octogenarian population demonstrated alarming progression, with 85% classified as high risk or functionally compromised.

### 4.2 The result of the interview analysis

**Figure 3:**
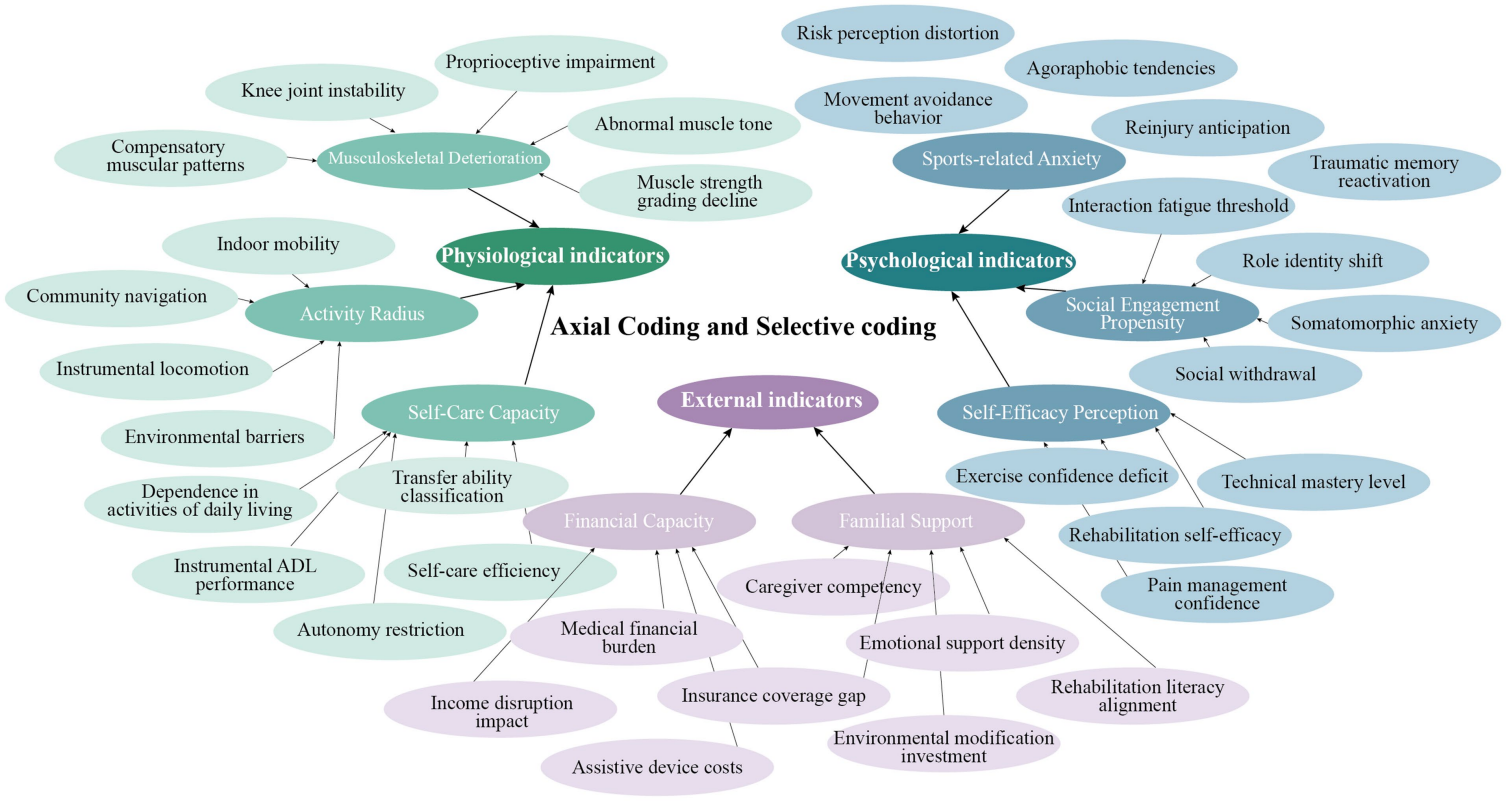
Axial coding and selective coding of the interview (draw by author).

Open coding identified 68 initial concepts through line-by-line annotation, generating nodes like “Decline in knee joint stability” and “Increased dependency in activities of daily living (ADL)” (Supplementary Table S3). Axial coding established relational hierarchies using NVivo’s matrix coding, linking categories, such as connecting “proprioceptive impairment” to “agoraphobic tendencies” through causal chains. Selective coding systematically integrated three core dimensions: physiological (musculoskeletal degeneration, activity radius, self-care capacity); psychological (Sports-related anxieties, self-efficacy perception and social engagement propensity); external (financial capacity and familial support) (Figure 3). This iterative process synthesized a tripartite framework elucidating dynamic interactions among physiological, psychological, and social determinants in the progression of sarcopenia among aging populations.

**Figure 4:**
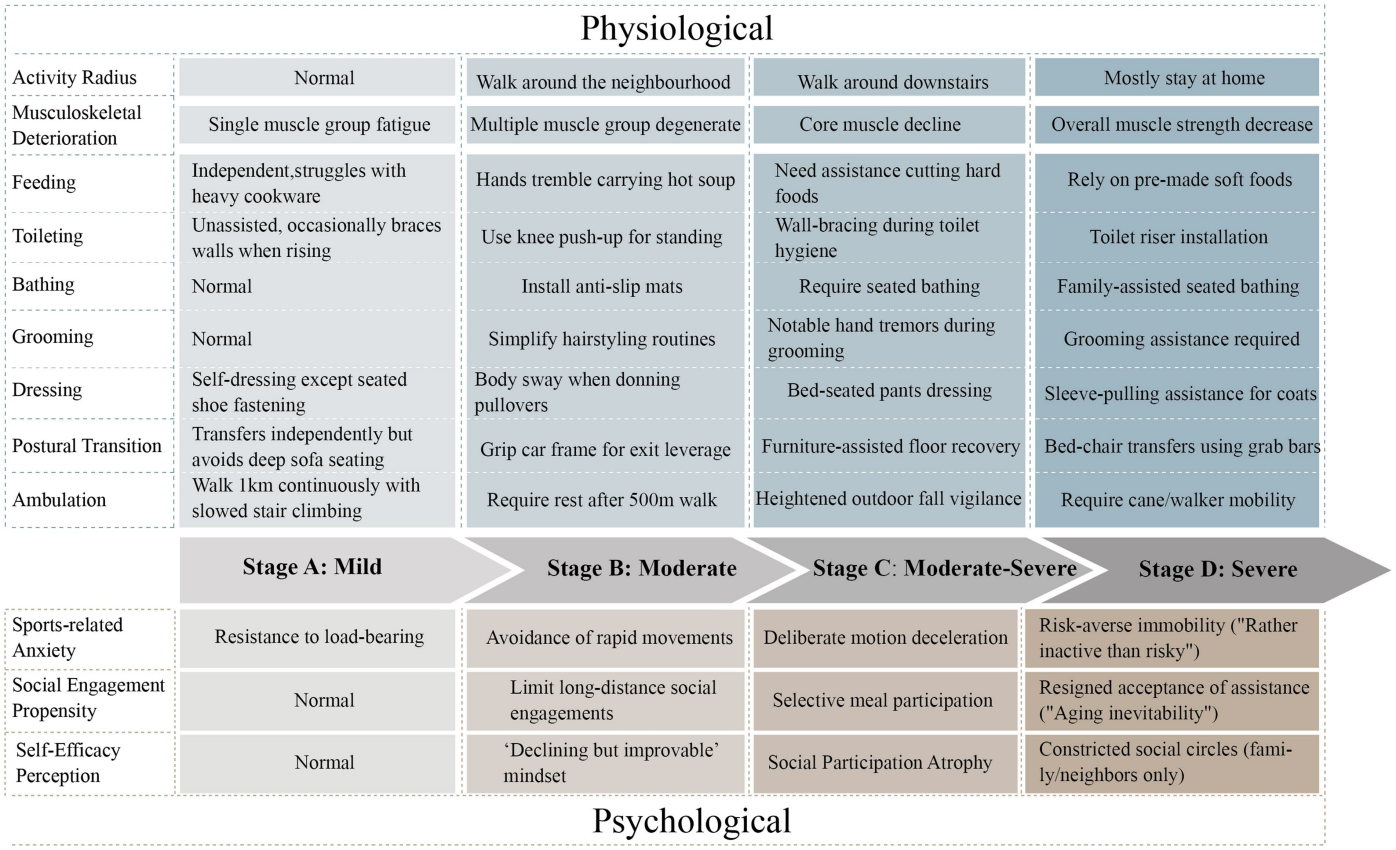
Patient reported muscle strength loss trajectory mapping (draw by author).

From the synthesized interview data, we categorized the target population into four clinically distinct progression stages (Figure 4). Stage A mild is defined as the Functional Compensation Phase characterized by an unrestricted mobility range complete independence in basic Activities of Daily Living and the presence of single muscle group fatigue manifestations such as difficulty lifting heavy objects with psychological features showing no movement avoidance behavior and normal social participation as captured in interview quotes occasionally tired but recovers quickly. Stage B moderate is defined as the Functional Compensation Attenuation Phase characterized by a mobility range reduced to the community level instrumental Activities of Daily Living requiring adaptive strategies like simplifying household chores and signs of multi-muscle group functional decline such as hand tremors while holding a bowl with psychological features revealing situational movement anxiety and selective engagement in social activities reflected in interview quotes choosing only flat paths when going out. Stage C moderate to severe is defined as the Functional Decompensation Phase characterized by a mobility range confined to the vicinity of the residence basic Activities of Daily Living requiring partial assistance such as help with chopping vegetables and signs of core muscle group deterioration such as needing to brace against walls when standing with psychological features indicating persistent movement fear and active social withdrawal evident in interview quotes afraid of falling avoids going out. Stage D severe is defined as the Functional Dependence Phase characterized by mobility restricted within the home dependence on assistance for basic Activities of Daily Living such as requiring accompaniment during bathing and signs of systemic muscle strength loss such as needing support to stand with psychological features demonstrating movement behavioral freezing and social circle collapse as expressed in interview quotes sitting feels safest. The staging framework fundamentally constitutes a clinical behavioral classification system.

To explicitly address potential equity concerns and ensure the framework’s applicability across diverse socioeconomic backgrounds, we employed stratified sampling based on Socioeconomic Status (SES). We utilized a scoring method to assess indicators of household income, personal education level, and occupation (based on pre-retirement status for retirees), in accordance with established SES classifications from scholars including Luo et al. (34) and An et al. (35). Each indicator was scored on a scale of 1 to 3 based on the criteria outlined in Supplementary Table S4. The total SES score ranged from 3 to 9, and participants were categorized into three distinct SES groups: Low SES: Total Score = 3–4; Medium SES: Total Score = 5–6; High SES: Total Score = 7–9.

From the full cohort, we systematically selected a validation subsample of 15 older adults (5 per SES group). Stage assignment was validated through methodological triangulation of self-assessed stage, SARC-F questionnaire and DXA assessment (Supplementary Table S5). Consistent staging accuracy across all three SES groups confirms the framework’s robustness against socioeconomic disparities, fulfilling its equitable design objective.

### 4.3 The result of the AHP analysis

Informed by our empirical findings, we developed a dynamic weight-shifting evaluation model (Table 5), in which Grade I interventions are dominated by physiological indicators (70% weighting) and as interventions escalate to Grade III, psychological metrics gain progressively higher priorities. Based on this framework and expert input through pairwise comparisons, we constructed the underlying judgment matrices. The consistency of these expert judgments was rigorously validated, with results shown in Supplementary Table S6. All consistency ratios (CR) were below 0.1, confirming the reliability of the expert inputs. Subsequently, integrating the validated judgments with the dynamic weighting framework, we derived the finalized, intervention-grade-specific weight sets, shown in Table 6.

**Table 5:** Weight allocation for disease progression intervention stratification.

Progression stage	Physiological indicators weight	Psychological indicators weight	External factors weight	Intervention grade
Stage A	70%	20%	10%	Grade I
Stage B	65%	25%	10%	Grade I/II
Stage C	60%	30%	10%	Grade II
Stage D	55%	35%	10%	Grade III

**Table 6:** Weight allocation and prioritization across indicator levels.

Stage	Primary indicators	Secondary indicators	Tertiary indicators	Composite weight	Global ranking
Stage A	Physiological dimension	Musculoskeletal deterioration	–	0.225	2
		Activity radius	–	0.086	4
		Self-care capacity	Postural transition	0.058	1
			Ambulation	0.058	
			Toileting	0.058	
			Dressing	0.039	
			Feeding	0.078	
			Bathing	0.058	
			Grooming	0.039	
	Psychological dimension	Sports-related anxiety	–	0.108	3
		Social engagement propensity	–	0.059	6
		Self-efficacy perception	–	0.033	7
	External factors	Familial support	–	0.075	5
		Financial capacity	–	0.025	8
Stage B	Physiological dimension	Musculoskeletal deterioration	–	0.209	2
		Activity radius	–	0.080	4
		Self-care capacity	Postural transition	0.054	1
			Ambulation	0.054	
			Toileting	0.054	
			Dressing	0.036	
			Feeding	0.072	
			Bathing	0.054	
			Grooming	0.036	
	Psychological dimension	Sports-related anxiety	–	0.135	3
		Social engagement propensity	–	0.074	6
		Self-efficacy perception	–	0.041	7
	External factors	Familial support	–	0.075	5
		Financial capacity	–	0.025	8
Stage C	Physiological dimension	Musculoskeletal deterioration	–	0.193	2
		Activity radius	–	0.074	6
		Self-care capacity	Postural transition	0.050	1
			Ambulation	0.050	
			Toileting	0.050	
			Dressing	0.033	
			Feeding	0.067	
			Bathing	0.050	
			Grooming	0.033	
	Psychological dimension	Sports-related anxiety	–	0.162	3
		Social engagement propensity	–	0.089	4
		Self-efficacy perception	–	0.049	7
	External factors	Familial support	–	0.075	5
		Financial capacity	–	0.025	8
Stage D	Physiological dimension	Musculoskeletal deterioration	–	0.177	3
		Activity radius	–	0.068	6
		Self-care capacity	Postural transition	0.046	1
			Ambulation	0.046	
			Toileting	0.046	
			Dressing	0.031	
			Feeding	0.061	
			Bathing	0.046	
			Grooming	0.031	
	Psychological dimension	Sports-related anxiety	–	0.189	2
		Social engagement propensity	–	0.104	4
		Self-efficacy perception	–	0.057	7
	External factors	Familial support	–	0.075	5
		Financial capacity	–	0.025	8

Analysis of the finalized weights in Table 6 revealed several key findings: Self-care capacity emerged as the dominant physiological evaluation dimension, with tertiary indicators *D*1 (basic mobility) and *D*6 (task persistence) proving critical for early sarcopenia detection. Movement-related anxiety ranked consistently highly (2nd or 3rd) across the psychological metrics, demonstrating a strong correlation with International Falls Efficacy Scale (FES-I) scores, thus constituting a pivotal intervention design focus. Musculoskeletal deterioration maintained elevated weighting throughout the progression stages, underscoring the irreversible nature of sarcopenia muscle loss and the necessity for assistive device integration and iterative optimization.

Finally, The algorithm commences with the Stage Scoring phase, taking the individual’s assigned stage as input to automatically retrieve the corresponding weight set. Specifically activating a 70% weight for physiological indicators in Stage A, a 65% weight in Stage B, with Stages C and D following analogous weighting schemes based on progression. It then proceeds to the Indicator Quantification phase, where individual indicators within each progression stage (A–D) are rated on a severity scale of 1 to 4 points by mapping behavioral markers from the stage models to validated clinical assessment tools: physiological indicators are scored using the SARC-F screening tool and the independence level derived from the Activities of Daily Living (ADL) scale, while psychological indicators are quantified by integrating the Falls Efficacy Scale-International (FES-I) for movement anxiety, the Hospital Anxiety and Depression Scale (HADS) for social participation tendency, and the General Self-Efficacy Scale (GSES) for psychological resilience. The final Dynamic Calculation phase computes the Total Intervention Score using the formula *Σ* (Indicator Score × Corresponding Stage Weight), which is then mapped to the appropriate intervention grade.

The stratification formula was defined as follows:


$${\text{Stage}}_{X}=\underset{\text{Physiological score}}{\underset{︸}{\sum_{\begin{array}{c} \text{Physiological}\;\\ \text{indicators} \end{array}}S_{i}\cdot w_{i}}}+\underset{\text{Physiological score}}{\underset{︸}{\sum_{\begin{array}{c} \text{Psychological}\\ \text{indicators} \end{array}}S_{j}\cdot w_{j}}}+\underset{\text{external score}}{\underset{︸}{\sum_{\begin{array}{c} \text{External}\\ \text{indicators} \end{array}}S_{k}\cdot w_{k}}}$$

**Figure 5:**
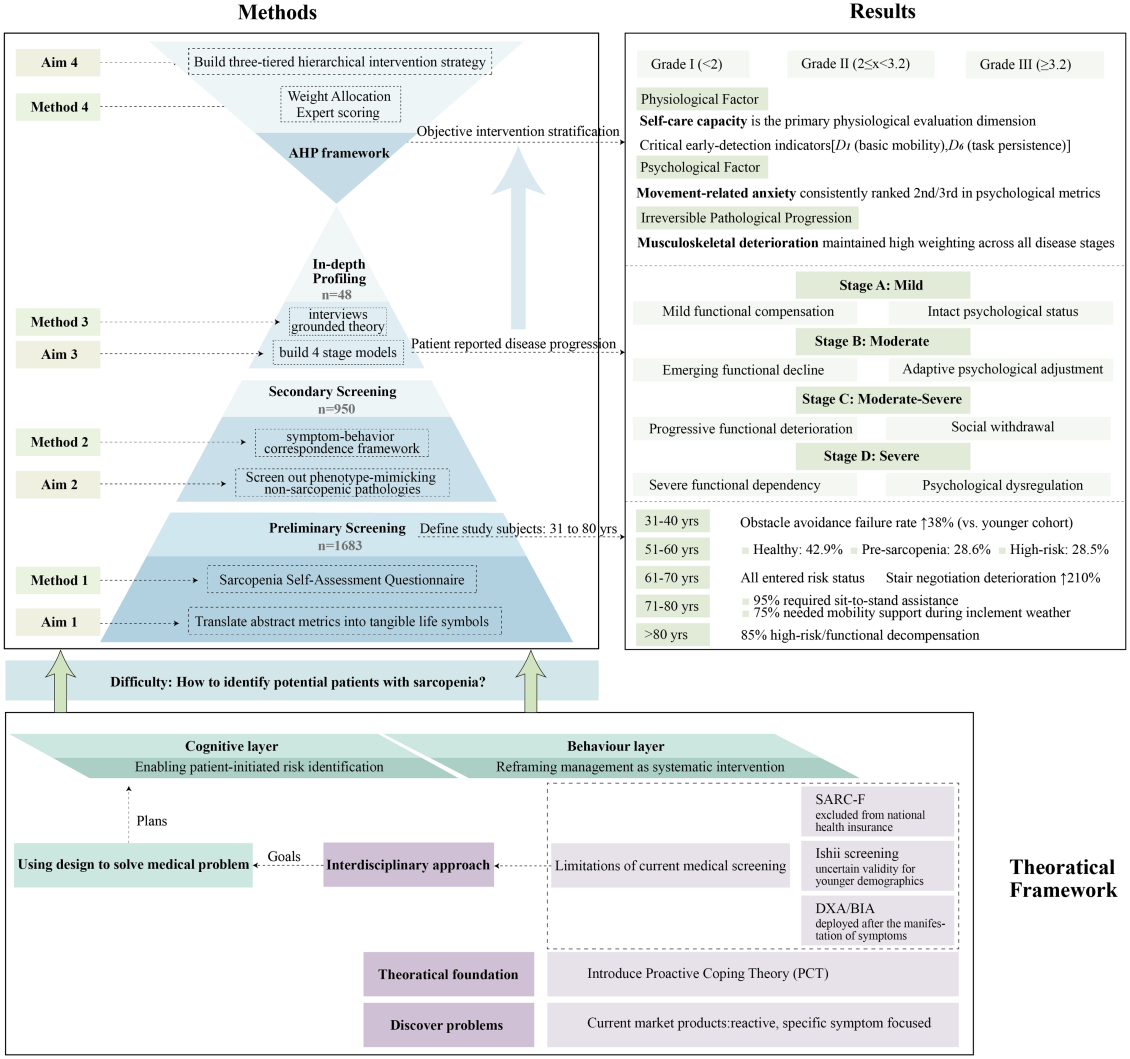
Integrative research framework for sarcopenia intervention development (draw by author).

Grade I interventions were recommended for scores of less than 2, Grade II interventions were applied to scores of between 2 and 3.2, and Grade III interventions corresponded to scores of over 3.2. Figure 5 delineates the translational operationalization of the Proactive Coping Theory in sarcopenia management into a tiered intervention workflow.

### 4.4 Computational tools and software implementation

This study employed a multi-method analytical approach to address distinct research dimensions. Quantitative analysis commenced with 1,683 questionnaire datasets using SPSS 27.0. The analytical sequence initiated with reliability and validity assessments, followed by the transformation of continuous age variables into six clinically significant age cohorts (≤30 to ≥81 years). Building upon this cohort stratification, the study computed distribution characteristics of muscle function parameters across age strata.

After that, we employed NVivo 14 software to conduct grounded theory analysis of 48 semi-structured interviews. Following verbatim transcription of interview texts, systematic open coding was performed using NVivo, which encompassed participant-derived concepts such as “knee joint instability” and researcher-constructed analytical concepts like “role identify shift.” Subsequent axial coding utilized NVivo’s matrix coding function to establish hierarchical relationships among categories, identifying causal pathways through systematic relational mapping. During the selective coding phase, iterative conceptual integration was facilitated by NVivo, consolidating nodes into three core dimensions. This analytical process was augmented by NVivo’s cluster analysis tools to identify attribute patterns across texts, ultimately deriving a data-driven four-stage clinical progression model, ranging from Stage A to Stage D.

Finally, AHP analysis was executed via Expert Choice 11.0 software. The hierarchical framework for sarcopenia intervention stratification was first constructed in the software’s Hierarchy Editor module. Subsequently, the judgment matrices from 10 experts were input level-by-level through the Judgment Matrix interface. The critical consistency validation phase was automatically executed by the system which aggregated expert data using the geometric mean method while calculating the Consistency Ratio (CR) for each hierarchy in real-time. To accommodate dynamic weighting requirements, four disease stage (A-D) were established in the Scenario Manager module. In Stage A, physiological factors were locked at 70% weighting priority. As scenarios progressed toward Stage D, psychological factors were incrementally prioritized, resulting in the weighting coefficient for exercise anxiety increasing from 0.108 to 0.189. Table 6 outputs the global weighting ranking of tertiary indicators, and documents coefficient variations across stages. The exported data was then embedded into the Latex algorithm to calculate intervention grades (I-III).

## 5 Discussion

### 5.1 Key findings and implications

Analytical findings confirm escalating sarcopenia risk with age progression, revealing two critical epidemiological patterns: Firstly, the emergence of pre-sarcopenia markers in adults aged 31–40 suggests modern sedentary lifestyles may accelerate sarcopenia pathogenesis, indicating a critical window for preventive interventions; Secondly, the observed divergence from Proactive Coping Theory in septuagenarians, compounded by comorbidities, necessitated clinical interventions beyond self-management. Consequently, we focused on respondents aged 31–80 years with functional scores of 1–24, as this cohort encompasses the full pathological spectrum while remaining amenable to self-directed interventions which is a key target for scalable public health strategies.

The four-stage classification model elucidated sarcopenia’s quantitative to qualitative pathological trajectory, providing a structured framework for subsequent intervention designs. This method enables early self-screening and risk stratification, empowering under-served individuals to proactively identify sarcopenia precursors before irreversible functional decline occurs.

**Figure 6:**
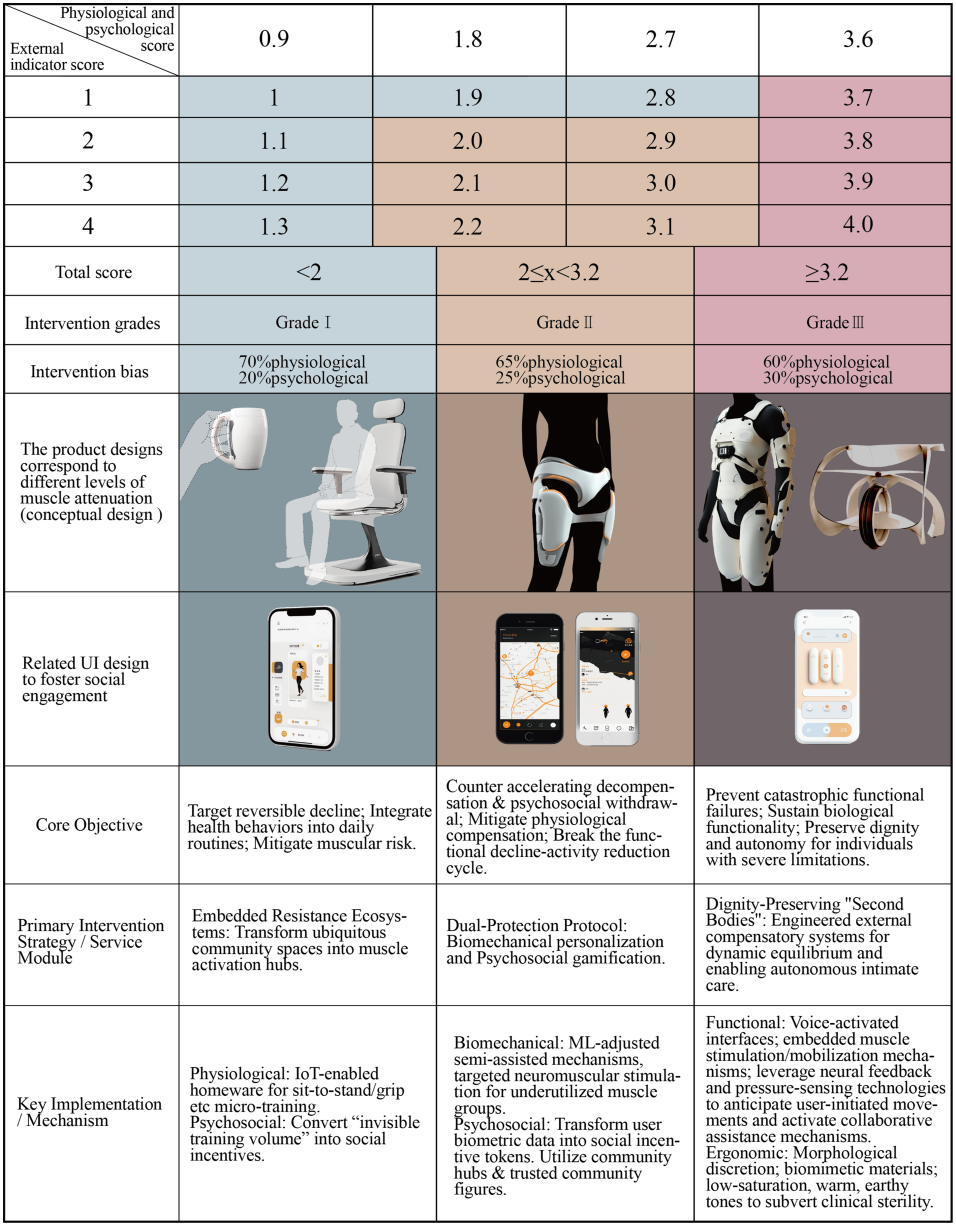
Score, intervention level and corresponding design (draw by author).

To resolve the paradox of inflated early-stage scores misaligning with clinical phenotypes, we instituted stage-constrained scoring with fixed 10% weighting for external factors (Table 5). This calibration ensures precise alignment between composite scores and intervention tiers:

Grade I interventions were recommended for scores of less than 2, targets reversible decline through muscular risk mitigation; Grade II interventions were applied to scores of between 2 and 3.2, counters accelerating decompensation and psychosocial withdrawal; and Grade III interventions corresponded to scores of over 3.2, prevents catastrophic failures in balance and psychosomatic function. The final composite scores, intervention tiers, and corresponding product design specifications are shown in Figure 6.

Rooted in Proactive Coping Theory’s “prevention first” principle, this three-tiered architecture operationalizes WHO Healthy Aging priorities by synchronizing individual behavioral change with community-level ecosystem engineering, transforming theoretical prevention-care continua into actionable population health strategy.

However, it must be stated that while the diagnostic staging model has been validated through triangulation, the three-tier intervention framework remains a conceptual proposition. Its overall effectiveness and feasibility requires future empirical validation. This research finalizes the theoretical construct phase encompassing problem diagnosis, demand stratification, and solution formulation. Consequently, empirical verification of solutions and quantitative efficacy assessment emerge as critical imperatives for subsequent investigation.

### 5.2 Grade I: prevention-centric intervention

The core strategy of Grade I interventions is aimed at the seamless integration of health behaviors into daily routines.

From a functional perspective, resistance exercises are considered one of the best ways to prevent sarcopenia and have been proven to reduce age-related muscle loss by over 50% (28). The central objective of this strategy would be to naturally embed resistance training into habitual daily movements, thereby potentially maximizing muscle growth during youth, stabilizing muscle mass maintenance during middle age, and minimizing age-related muscle atrophy in older adulthood. Given the extensive network of urban community dining halls established across China, with a national count reaching seventy-five thousand facilities (36), exemplified by Hangzhou City where community coverage exceeds 90 % (37), and serving diverse populations including older adults, migrant workers, and local residents, we propose exploring the transformation of these highly accessible public spaces into embedded, barrier-free muscle activation hubs. The envisioned approach involves conceptualizing an IoT-enabled preventive ecosystem to convert static dining behaviors into dynamic micro-training opportunities. As shown in Figure 6: left, through the functional modification of key environmental elements within community dining halls, such as chairs designed for sit-to-stand resistance training and utensils enabling grip strength exercises, could allow older adults naturally engage key muscle groups during their routine meals. This modality is intended to circumvent the psychological burden associated with traditional weight training while aiming to optimize muscle retention efficacy across the lifespan.

From a technological perspective, wearable sensors with EMG biofeedback could potentially dynamically adjust resistance parameters and enable the precise targeting of muscle groups, as well as encouraging sustainable health maintenance.

From an aesthetic perspective, by adhering to sociopsychological desensitization principles, designs can adopt consumer electronics-inspired CMF (color, material, and finish) languages to minimize medical stigma.

Observing the social connectivity dimension, a proposed system could automatically captures and records “invisible training volume” completed during meals. This data then be translated into tangible incentives within the community dining hall, such as meal discounts or redeemable community service credits. This envisioned localized, concrete positive feedback mechanism is designed to effectively drive the spontaneous formation and diffusion of health behaviors among high-risk populations.

The proposed innovative breakthrough of the Grade I intervention design strategy lies in its fundamental reconceptualization of the resistance training paradigm. This paradigm defines it as a dual ecosystem integrating biomechanical factors, manifested through the synergy of body, object, and environment, and sociocultural factors, characterized by the interweaving of individual, community, and emotional permeation. Crucially, the envisioned model possesses an inherently inclusive nature, equipping it to potentially serve urban older adults across varying socioeconomic status strata, with particular benefit for vulnerable groups such as those living alone, possessing low digital literacy, or experiencing mobility limitations. Simultaneously, by being designed to deeply embedding within the essential daily context of mealtimes, the strategy aims to ensure core characteristics of high frequency and low burden, ultimately aspiring to transform muscle maintenance into a sustainable daily habit as natural as daily meals.

### 5.3 Grade II: augmentation-focused intervention

The core strategy of Grade II interventions aims to mitigate physiological compensation that stems from sarcopenia progression. This tier proposes a dual-protection protocol designed to balance biomechanical and psychosocial factors to disrupt the negative functional decline-activity reduction cycle. On a physiological optimization level, the primary objectives focus on reducing skeletal compensation to mitigate exercise-induced comorbidities. Given the critical weight-bearing role and high-frequency utilization of the knee joint, it is predisposed to the early-onset irreversible degeneration; therefore, semi-assisted mechanisms could be engineered through biomechanical personalization. This concept involves machine learning-driven adjustment of resistance parameters based on real-time EMG patterns and joint kinematics, combined with targeted neuromuscular stimulation of underutilized muscle groups. Such integration is theorized to counteract motor function deterioration from prolonged compensatory reliance while preserving residual musculoskeletal integrity. Concurrently, targeted neuromuscular stimulation applied to underutilized muscle groups is proposed to counteracting motor function deterioration from prolonged compensatory reliance and preserving residual healthy musculoskeletal integrity (Figure 6: center panel).

At the psychosocial reinforcement level, we deeply acknowledge that socioeconomic status represents a critical social determinant of health outcomes for older adults (38). Research unequivocally demonstrates that lower socioeconomic status directly correlates with accelerated aging trajectories and a heightened prevalence of various health issues (39). Crucially, social engagement plays a significant yet incomplete mediating role in the pathway through which socioeconomic status impacts the physical and mental well-being of the older adults (40). Consequently, ensuring equitable accessibility and effectiveness for older adult populations across diverse socioeconomic strata has been a foundational design priority for this initiative from its inception. Addressing the critical challenge of effectively motivating older adults with lower socioeconomic status to overcome barriers and actively participate in social activities is central to the conceptual framework.

To address this challenge, the design proposes employing behavioral gamification protocols. This approach would transform user biometric data into social incentive tokens. These tokens could manifest as features like geolocation-based route sharing capabilities and exercise credit systems redeemable for essential community services. Examples include vouchers for daily necessities or discounts at community canteens. To minimize participation barriers, the program would strategically utilizes familiar and readily accessible community spaces as social hubs. Examples include community activity centers, neighborhood health service stations, and common areas within residential complexes. Simultaneously, the concept envisions actively empowers existing trusted community figures. These individuals would serve as activity organizers and trust mediators, leveraging established social networks to effectively reduce psychological obstacles and the trust cost associated with participation. Activity design would consciously avoids complex rules or high technical demands, instead advocating for natural interactions grounded in everyday life. The resulting latent psychosocial is intended to support framework continuously delivers positive behavioral reinforcement. It is designed to guide users toward cognitively reframing mobility, shifting its perception from passive deficit mitigation toward autonomy-driven empowerment.

Furthermore, specifically addressing the unique biopsychosocial profile of the 51 to 60-year-old cohort, the proposed design of outdoor assistive devices strictly adheres to contextual ergonomic principles. It incorporates streamlined forms devoid of superfluous ornamentation and integrates nocturnal illumination functionality. These features are intended to ensure optimal adaptation to common scenarios such as daily shopping trips and recreational walks. Complementing this design, vitality-enhancing color schemes would be implemented with the specific aim of alleviating anxiety stemming from awareness of somatic functional decline.

This tier conceptually achieves the symbiotic advancement of musculoskeletal optimization and social agency restoration via technological mediation and social infrastructure reengineering.

### 5.4 Grade III: protection-prioritizing intervention

The core strategy of Grade III interventions is constructing dignity-preserving “second bodies” that are engineered to sustain biological functionality and uphold the dignity of individuals with severe physical limitations. At its foundational level, this tier prioritizes dynamic equilibrium compensation, integrating compensatory force mechanisms to mitigate secondary injuries caused by falls. In addressing advanced functionalities, the target demographic, characterized by a heightened dependency on assistive tools and caregivers, the near-total loss of self-care capacity, and severely restricted mobility, demands solutions that transcend conventional wearable devices and extend toward external compensatory systems that enable limited autonomous movement. Consequently, these interventions require rigorous attention to functional precision and ergonomic comfort. Firstly, recognizing the decline in manual dexterity among users, these systems should incorporate voice-activated interfaces to streamline interactions. Secondly, acknowledging the long-term consequences of prolonged static postures, including muscular rigidity and atrophy, these designs should integrate embedded muscle stimulation and mobilization mechanisms. These aim to counteract the physical deterioration associated with prolonged immobility, thereby enhancing users’ somatic well-being. Thirdly, leveraging neural feedback and pressure-sensing technologies, these systems should anticipate user-initiated movements and activate collaborative assistance mechanisms. This synergy could empower users to independently perform intimate daily tasks, such as bathing and toileting, while preserving their agency as the primary initiators of motion rather than passive subjects of mechanical controls. By foregrounding user autonomy and physiological integrity, these interventions can not only address functional deficits but also reinforce user identity as dignified, socially engaged individuals (Figure 6: right panel).

These approaches could ensure the preservation of fundamental self-care capacities within safe parameters, while actively combating disease-related stigmas. The integration of physiological safeguarding and dignity preservation establishes a framework for holistic user empowerment, in which functional autonomy and social identity are synergistically maintained.

Aesthetically, these designs should acknowledge potential patient resistance to overtly medicalized devices, prioritizing morphological discretion to minimize their visual presence. Materiality should adhere to biomimetic principles, incorporating flexible exoskeletal structures and natural organic textiles to emulate biological continuity. Chromatically, palettes of low-saturation, warm, earthy tones are suggested to subvert the clinical sterility that is typically associated with medical environments.

From a public health intervention perspective, Grade I interventions activate preventive consumption markets via the convergence of fitness technologies and smart home ecosystems, establishing circular resource synergies between personal health data and domestic energy flows. Grade II interventions cultivate product–service symbiosis by hybridizing age-adaptive product designs with community-based care networks, creating resource-efficient older adults care systems. Grade III interventions optimize temporal resource allocation through regenerative workforce development, converting familial care into productive economic participation.

Through a social determinants of health framework, Grade I interventions constitute preventive health infrastructure via context-embedded resistance training, fostering behavioral resilience across generations. Grade II interventions advance circular longevity narratives by reframing aging as phased capacity adaptations, dismantling linear age stereotypes using intergenerational solidarity frameworks. Grade III interventions embody universal design ethics by enabling autonomous intimate care, constructing human-centered rehabilitation ecosystems that maintain social agency.

Within sustainable welfare systems, Grade I interventions extend independent living throughout life stages via primary prevention systems that are aligned with lifespan-related healthcare paradigms. Grade II interventions implement capacity-preserving innovations that delay functional decline, while reinforcing regenerative healthcare values. Grade III interventions deploy failsafe environmental designs to minimize fall risks, establishing dependency-reduction frameworks that preserve personal efficacy via adaptive assistive ecosystems.

### 5.5 Implementation considerations and limitations

However, the generalizability and practical implementation of our findings and the proposed tiered intervention framework face significant challenges, with a core limitation rooted in an “urban-centric” perspective.

Firstly, the applicability of our results to broader populations is constrained by the urban socioeconomic context of the study cohort. Participants were recruited exclusively from tier-1 and tier-2 Chinese cities, where healthcare infrastructure, insurance profiles and living environments differ substantially from rural settings. Key characteristics of this urban bias include: Over-representation of Employee Basic Medical Insurance (EmpIns), covering 45% of our sample compared to the national coverage of 26.2% (41); Under-representation of the New Rural Cooperative Medical Scheme (NRCMS) and rural older adults, our cohort contained only 16.3% of individuals aged ≥ 60 years, significantly below the rural proportion of 23.81% (42); and higher average education levels, 10.67 years versus the national average of 9.91 years (43). Furthermore, small subgroup sizes (e.g., *n* = 2 in the 31–40y D-Prof group with 100% EmpIns coverage) may introduce selection bias. The questionnaire’s reliance on urban-centric symbols like courier stations and living rooms for self-assessment descriptions of sarcopenia creates significant cognitive dissonance in rural contexts, rendering the risk perception tool difficult to apply effectively.

Rural older adults themselves face compounded vulnerabilities including poorer nutritional status, high widowhood rates and prevalence of left-behind status among older women, and lack of economic and emotional support. These factors lead to significantly higher risks of social isolation and depression compared to their urban counterparts (44). Beyond theoretical generalizability, the implementation of the tiered intervention framework itself encounters multiple obstacles due to this convergence of vulnerabilities.

For Grade I interventions, the high cost of Internet of Things (IoT) technology renders it largely unfeasible in rural settings. Moreover, within patriarchal cultural norms, older women engaging in resistance training within traditional, male-dominated household spaces like the central living hall may be perceived as transgressing spatial boundaries. Simultaneously, their unpaid, burdensome domestic labor is often tacitly viewed as an inherent duty, prioritizing personal health behaviors can be criticized as dereliction of duty or selfishness.

Critically, the accessibility of Grade II and III interventions is severely hampered by characteristics prevalent in the broader older adults population, especially outside our highly educated urban cohort. According to the Fifth National Sampling Survey on the Living Conditions of China’s Urban and Rural Older Adults, 61.0% of older adults Chinese have an educational attainment at or below elementary school level, with 37% lacking Mandarin proficiency – a demographic characteristic concentrated in the aging population. The digital literacy gap impedes engagement with advanced technologies, while voice-activated interfaces face linguistic exclusion due to inadequate dialect support in commercial Automatic Speech Recognition (ASR) systems. As most rural older adults rely on farming or casual labor to supplement their meager pensions, the cost of assistive solutions dependent on sophisticated equipment far exceeds the affordability threshold set by the Basic Pension Insurance for Urban and Rural Residents, making them economically unsustainable.

Despite these limitations, the study’s focus on an urban sample reflects China’s ongoing and profound urbanization process. Since the founding of the People’s Republic of China in 1949, the population has transformed from a predominantly rural society to an urbanized one. Consequently, the current population distribution is a direct outcome of this historical trajectory:

Aged 70–80 cohorts remain predominantly rural due to lower mobility. Middle-Aged Cohorts (40–60) exhibits high rates of rural-to-urban migration, primarily driven by employment opportunities and, increasingly, relocation to join adult children residing in cities. The massive scale of internal migration is underscored by the 2020 Census, which recorded 492.76 million internal migrants with a 69.73% increase since 2010 (45). Crucially, 375.82 million were classified as “floating population” (residing outside their registered Hukou location), including 124.84 million inter-provincial migrants (45); a substantial and growing proportion of Younger Cohorts (Under 40) consists of individuals born in urban settings, reflecting decades of urbanization. The 2020 Census confirmed that 63.89% (901.99 million people) now reside in urban areas, a significant increase from previous decades (45).

While the urban sample holds value for studying future societal structures under China’s urbanization trend, it cannot compensate for the lack of research focus on the currently most vulnerable populations. Therefore, the study’s conclusions and interventions, particularly for disadvantaged rural older adults with low education, dialect usage, and economic hardship, require rigorous localization and validation.

Consequently, future research must prioritize two missions: First, empirically validate our proposed conceptual framework. Initial studies should focus on assessing the feasibility and acceptability of implementing the framework’s core components, such as the IoT-enhanced proactive coping strategies for Grade I sarcopenia in real-world community settings. Subsequent research should evaluate the framework’s effectiveness in preventing sarcopenia progression and falls, and its impact on psychosocial outcomes across different intervention tiers. Long-term studies incorporating objective measures like DXA and health economic analyses will be crucial to determine the framework’s sustainability and value. Second, adapt interventions for rural older adults through reconstructing sarcopenia self-assessment tools using descriptions grounded in rural life contexts; exploring the feasibility of low-cost, core intervention pathways adapted to resource constraints, leveraging village doctor networks and utilizing everyday farming tools; advocating for the inclusion of contextually appropriate basic screening and early interventions within the New Rural Cooperative Medical Scheme (NRCMS) reimbursement framework to provide institutional support for implementation.

## 6 Conclusion

This study provides a replicable public health framework for sarcopenia management through proactive design methodologies. It aims to transcend the functional limitations of conventional rehabilitation products via non-medical health strategies, while concurrently addressing the emotional needs and social dignity of users. This approach aligns with the synergistic development of health management strategies and humanistic care under the paradigm of the silver economy.

Theoretically, the translation of proactive adaptation theory in this study manifested in two dimensions. The first dimension was proactive assessment, in which we transposed quantitative medical diagnostics into symbolic interpretations of daily living, rendering abstract muscular degeneration risks as perceptible embodied behaviors. This established a quantifiable formula that links subjective disease staging to objective intervention tiers. The second dimension was proactive intervention, in which we developed a three-tier strategic framework to empower individuals across sarcopenia severity levels to actively engage with their physiological and psychological changes, fostering their participation in societal co-construction.

Practically, the study followed a problem identification–theoretical translation–model construction–applied implementation workflow. Phase 1 used market analyses to reveal systemic inadequacies in sarcopenia-targeted products within aging societies. Phase 2 articulated the innovative value of proactive adaptation theory in reconfiguring reactive healthcare models. Phase 3 developed a life cycle intervention system using a dynamic stratified sampling analysis. Phase 4 delivered three graded intervention prototypes for real-world applications. Grounded in interdisciplinary sustainability science, this study positions design as a catalyst for systemic transition, operationalizing epistemic autonomy via a framework that synchronizes the biophysical preservation, socioeconomic regeneration, and cultural revalorization of aging populations.

## Data Availability

The original contributions presented in the study are included in the article/Supplementary material, further inquiries can be directed to the corresponding authors.

## Footnotes

^1^First-tier cities: Internationally recognized as global metropolises characterized by mega-scale urbanization and exceptional geopolitical influence in global economic networks. The current consensus identifies four Chinese first-tier cities: Beijing, Shanghai, Guangzhou, and Shenzhen. Second-tier cities are mostly provincial capitals or special economic zone hubs, which includes: Chengdu, Chongqing, Hangzhou, Xi’an, Wuhan, Suzhou, Zhengzhou, Nanjing, Tianjin, Changsha, Dongguan, Ningbo, Foshan, Hefei, and Qingdao, Fuzhou, etc.
