# Supplementary material for: Developing a proactive coping theory-based conceptual framework for sarcopenia management in aging societies: a mixed-methods study from China
Source: Front Public Health. 2025 Nov 20;13:1604370. doi: 10.3389/fpubh.2025.1604370 (PMC12675246; doi:10.3389/fpubh.2025.1604370)
Supplement: Supplementary file 1 [file Table_1.DOCX]

Supplementary Materials

**Supplementary Table S1.** Results of Reliability Analysis

| **Scale/Dimension** | **Cronbach's α** |
| --- | --- |
| Total Scale | 0.89 |
| Strength Performance | 0.85 |
| Endurance Capacity | 0.82 |
| Balance Regulation | 0.78 |
| Functional Adaptation | 0.81 |

**Supplementary Table S2.** Kaiser-Meyer-Olkin and Bartlett’s Test of Sphericity

| KMO Measure of Sampling Adequacy | | 0.92 |
| --- | --- | --- |
| Bartlett’s Test of Sphericity | Approximate Chi-Square | 8765.432 |
|  | Degrees of Freedom | 66 |
|  | Significance | 0.000 |

**Supplementary Table S3.** NVIVO open coding example

| **Interview content** | **Open coding examples** |
| --- | --- |
| "When I walk now, my knees feel weak all the time, and I have to hold onto the handrail when going upstairs." | Decline in knee joint stability |
| "I have to wait for my daughter to come home from work to help me with things like bathing and getting dressed." | Increased dependency in activities of daily living (ADL) |
| "I'm even scared to go to the supermarket now. If I fall again, it'll cost so much money." | Financial burden anxiety / Agoraphobic tendencies |
| "During physical therapy at the hospital, I keep remembering when I got injured last time. My heart pounds just thinking about it." | Traumatic memory reactivation / Insufficient exercise self-efficacy |
| "The neighbors invited me to square dancing, but with these legs of mine..." | Social avoidance / Somatomorphic anxiety |

**Supplementary Table S4.** Socioeconomic Status (SES) Scoring Criteria

| **Income** | **Education** | **Occupation** | **Scores** |
| --- | --- | --- | --- |
| ≥ 8000 | High School or Above | Managerial / Professional / Technical / Administrative Personnel | 3 |
| 4000 - 7999 | Junior High School | Service / Sales / Skilled Worker / Farmer | 2 |
| < 4000 | Primary School or Below | Unemployed / Inactive / Other Laborers | 1 |

**Supplementary Table S5.** triangular methodological validation results

| Patient ID | Stage | Sex | SARC-F | DXA-SMI | SES Group |
| --- | --- | --- | --- | --- | --- |
| PA-01 | A | Female | 2 | 6.0 | High |
| PB-02 | B | Female | 5 | 6.8 | High |
| PC-03 | C | Male | 7 | 5.6 | Low |
| PC-04 | C | Male | 7 | 5.5 | High |
| PD-05 | D | Male | 9 | 4.8 | Medium |
| PD-06 | D | Male | 9 | 5.1 | Medium |
| PC-07 | C | Female | 8 | 5.0 | Low |
| PD-08 | D | Female | 9 | 4.3 | Low |
| PD-09 | D | Female | 9 | 4.1 | Medium |
| PC-10 | C | Male | 7 | 5.5 | Medium |
| PB-11 | B | Male | 4 | 6.2 | High |
| PD-12 | D | Female | 8 | 4.4 | Medium |
| PA-13 | A | Male | 3 | 7.8 | Low |
| PB-14 | B | Male | 4 | 6.6 | High |
| PC-15 | C | Male | 6 | 6.0 | Low |

**Supplementary Table S6.** Consistency validation results

| Items | λmax | *CI* | *RI* | *CR* |
| --- | --- | --- | --- | --- |
| *B*1 | 3.036 | 0.018 | 0.58 | 0.031 |
| *B*2 | 3.029 | 0.014 | 0.58 | 0.024 |
| *B*3 | 2.000 | 0.000 | 0.000 | 0.000 |
